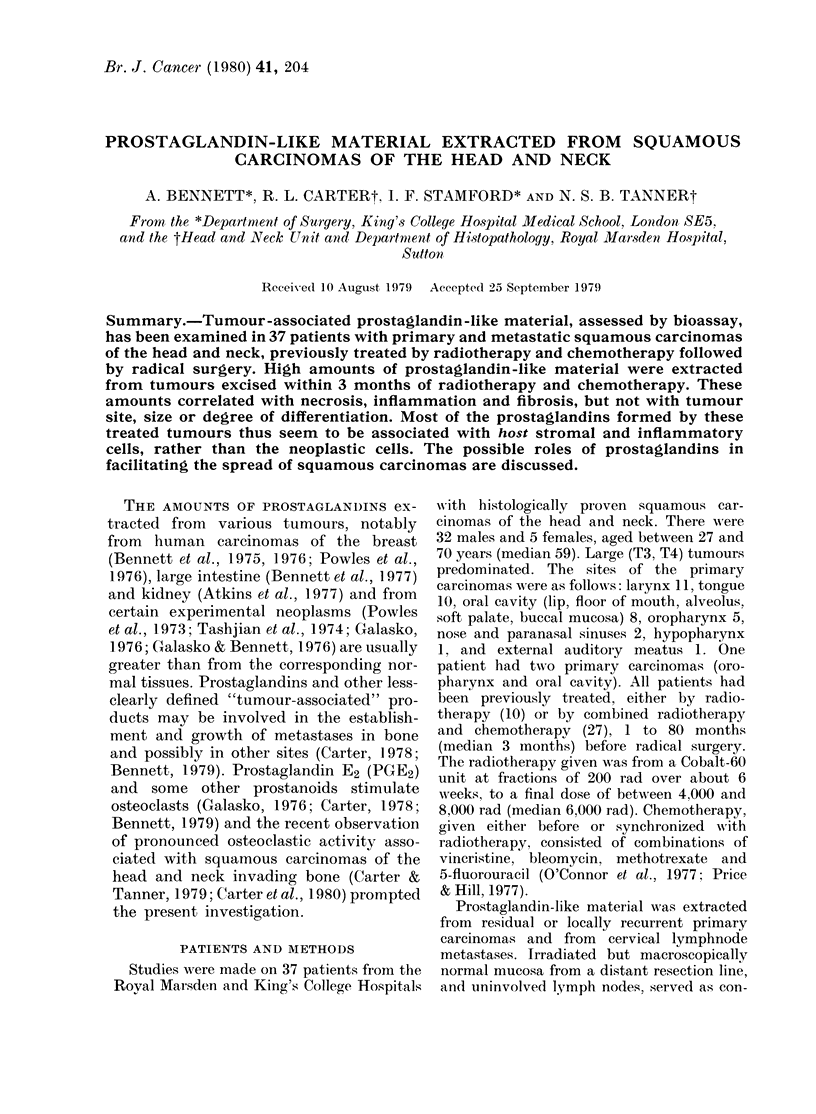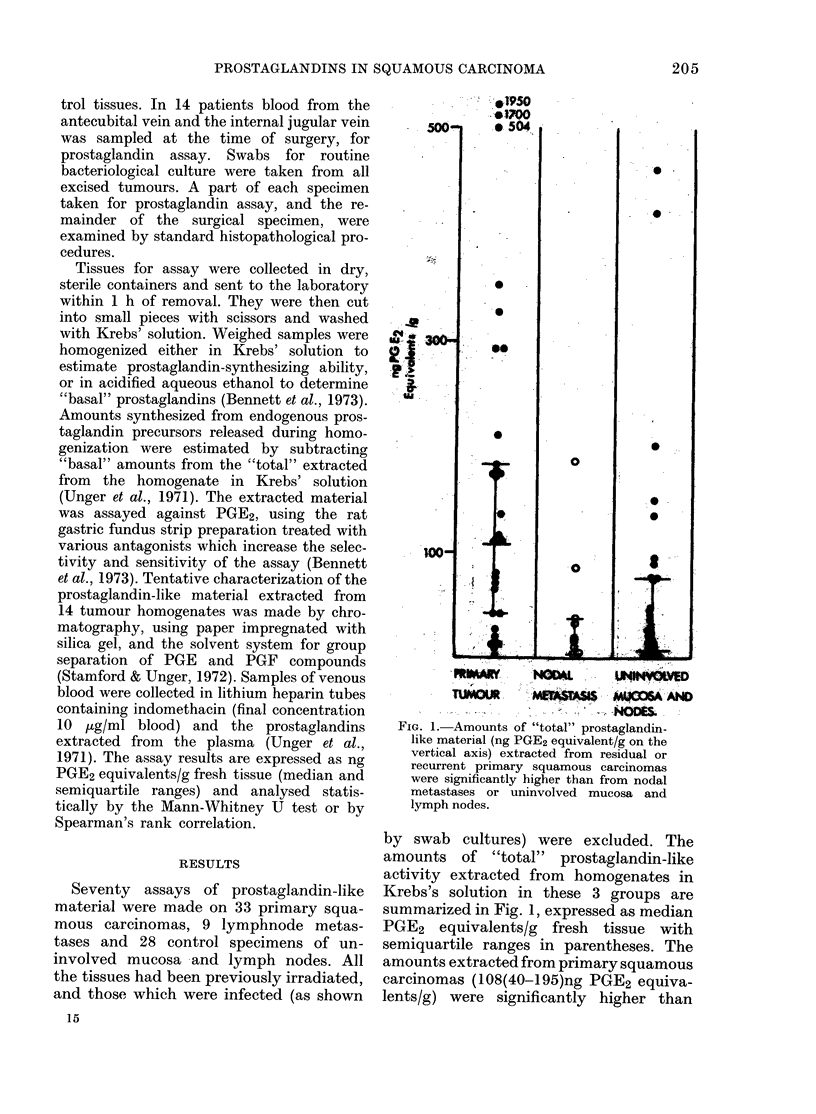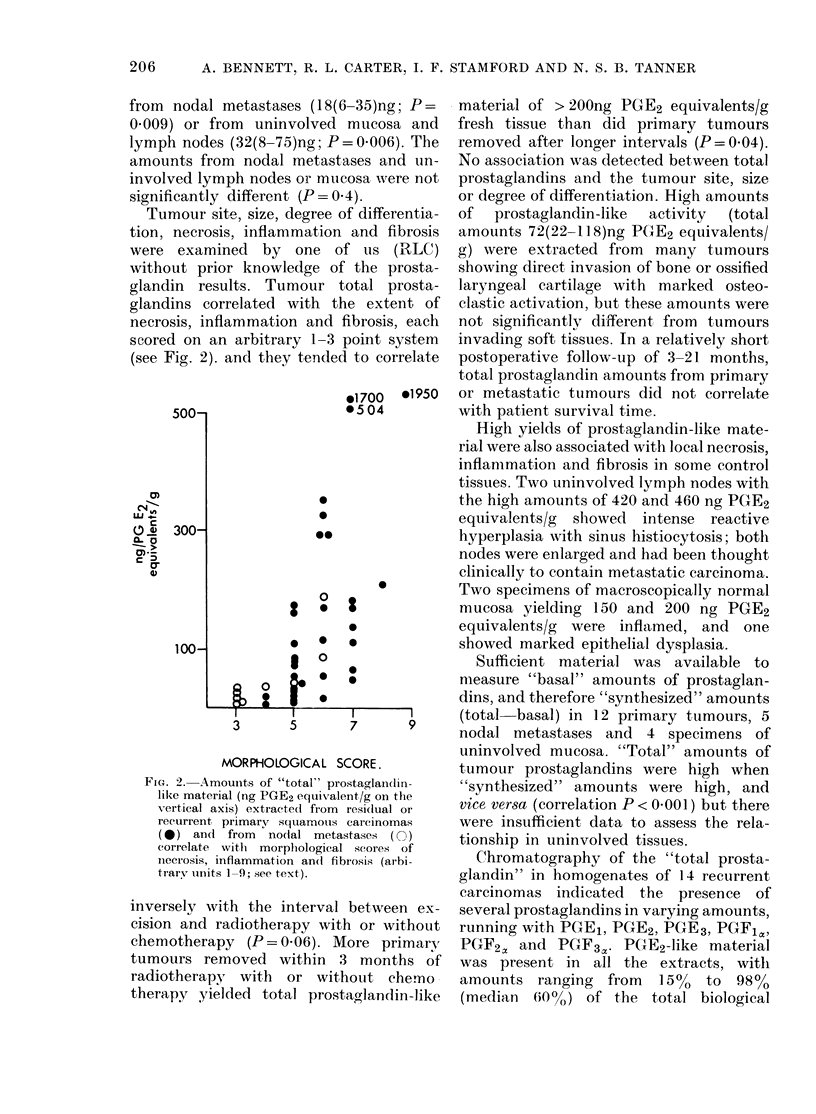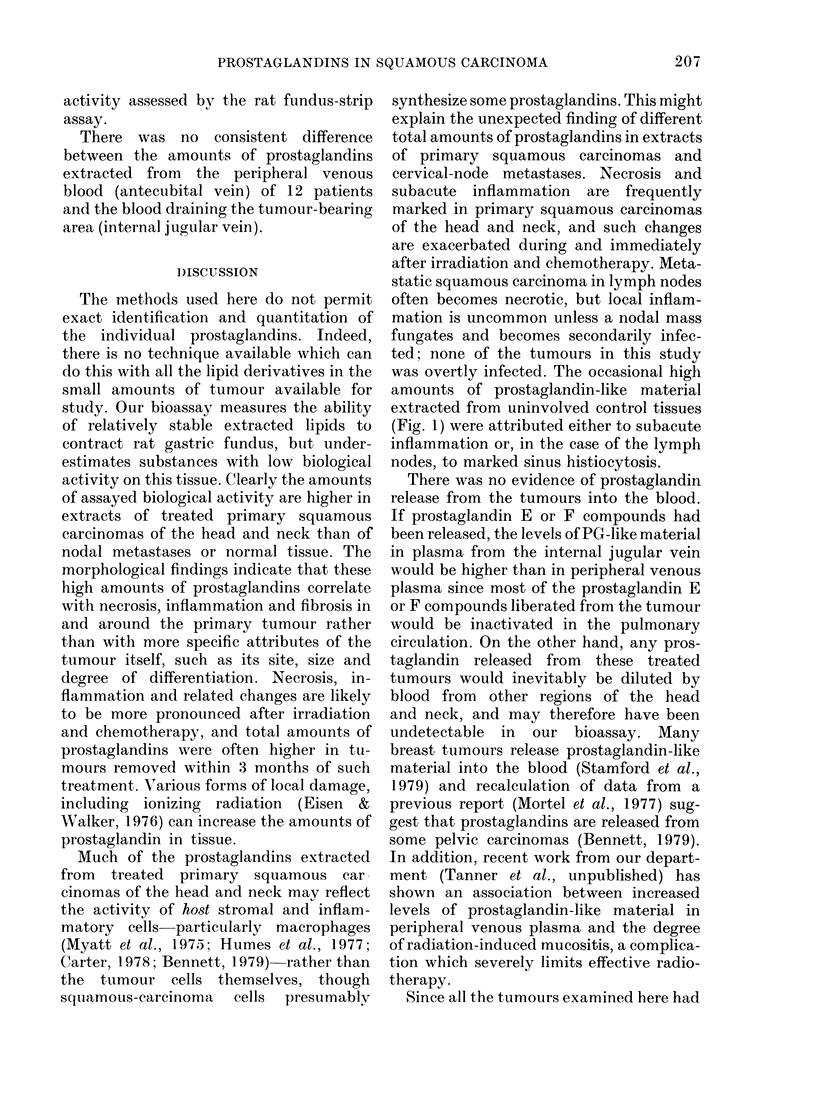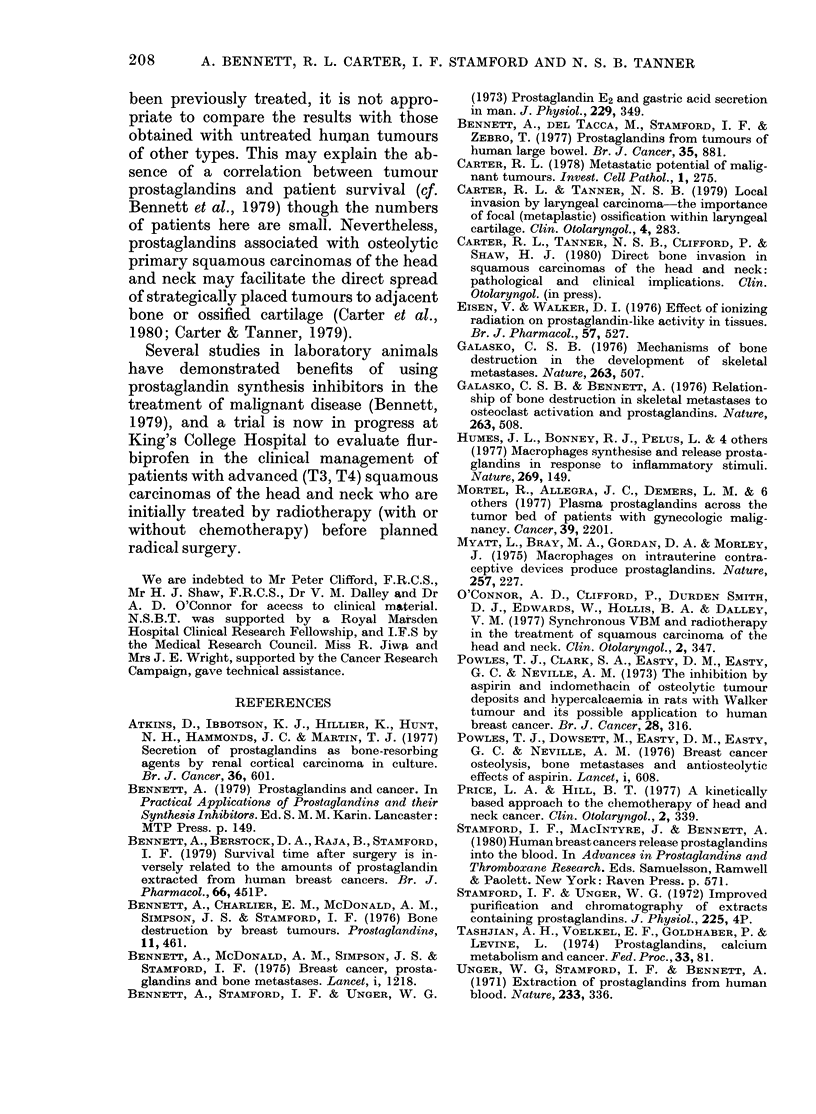# Prostaglandin-like material extracted from squamous carcinomas of the head and neck.

**DOI:** 10.1038/bjc.1980.31

**Published:** 1980-02

**Authors:** A. Bennett, R. L. Carter, I. F. Stamford, N. S. Tanner

## Abstract

Tumour-associated prostaglandin-like material, assessed by bioassay, has been examined in 37 patients with primary and metastatic squamous carcinomas of the head and neck, previously treated by radiotherapy and chemotherapy followed by radical surgery. High amounts of prostaglandin-like material were extracted from tumours excised within 3 months of radiotherapy and chemotherapy. These amounts correlated with necrosis, inflammation and fibrosis, but not with tumour site, size or degree of differentiation. Most of the prostaglandins formed by these treated tumours thus seem to be associated with host stromal and inflammatory cells, rather than the neoplastic cells. The possible roles of prostaglandins in facilitating the spread of squamous carcinomas are discussed.


*Br. J. Cancer* (1980) **41**, 204

### PROSTAGLANDIN-LIKE MATERIAL EXTRACTED FROM SQUAMOUS CARCINOMAS OF THE HEAD AND NECK

A. BENNETT*, R. L. CARTER†, I. F. STAMFORD* AND N. S. B. TANNER†

*From the *Department of Surgery, King's College Hospital Medical School, London SE5, and the †Head and Neck Unit and Department of Histopathology, Royal Marsden Hospital, Sutton*

Received 10 August 1979 Accepted 25 September 1979

**Summary.—Tumour-associated prostaglandin-like material, assessed by bioassay, has been examined in 37 patients with primary and metastatic squamous carcinomas of the head and neck, previously treated by radiotherapy and chemotherapy followed by radical surgery. High amounts of prostaglandin-like material were extracted from tumours excised within 3 months of radiotherapy and chemotherapy. These amounts correlated with necrosis, inflammation and fibrosis, but not with tumour site, size or degree of differentiation. Most of the prostaglandins formed by these treated tumours thus seem to be associated with *host* stromal and inflammatory cells, rather than the neoplastic cells. The possible roles of prostaglandins in facilitating the spread of squamous carcinomas are discussed.**

THE AMOUNTS OF PROSTAGLANDINS extracted from various tumours, notably from human carcinomas of the breast (Bennett *et al.*, 1975, 1976; Powles *et al.*, 1976), large intestine (Bennett *et al.*, 1977) and kidney (Atkins *et al.*, 1977) and from certain experimental neoplasms (Powles *et al.*, 1973; Tashjian *et al.*, 1974; Galasko, 1976; Galasko & Bennett, 1976) are usually greater than from the corresponding normal tissues. Prostaglandins and other less-clearly defined "tumour-associated" products may be involved in the establishment and growth of metastases in bone and possibly in other sites (Carter, 1978; Bennett, 1979). Prostaglandin $E_2$ ($PGE_2$) and some other prostanoids stimulate osteoclasts (Galasko, 1976; Carter, 1978; Bennett, 1979) and the recent observation of pronounced osteoclastic activity associated with squamous carcinomas of the head and neck invading bone (Carter & Tanner, 1979; Carter *et al.*, 1980) prompted the present investigation.

### PATIENTS AND METHODS

Studies were made on 37 patients from the Royal Marsden and King's College Hospitals with histologically proven squamous carcinomas of the head and neck. There were 32 males and 5 females, aged between 27 and 70 years (median 59). Large (T3, T4) tumours predominated. The sites of the primary carcinomas were as follows: larynx 11, tongue 10, oral cavity (lip, floor of mouth, alveolus, soft palate, buccal mucosa) 8, oropharynx 5, nose and paranasal sinuses 2, hypopharynx 1, and external auditory meatus 1. One patient had two primary carcinomas (oropharynx and oral cavity). All patients had been previously treated, either by radiotherapy (10) or by combined radiotherapy and chemotherapy (27), 1 to 80 months (median 3 months) before radical surgery. The radiotherapy given was from a Cobalt-60 unit at fractions of 200 rad over about 6 weeks, to a final dose of between 4,000 and 8,000 rad (median 6,000 rad). Chemotherapy, given either before or synchronized with radiotherapy, consisted of combinations of vincristine, bleomycin, methotrexate and 5-fluorouracil (O'Connor *et al.*, 1977; Price & Hill, 1977).

Prostaglandin-like material was extracted from residual or locally recurrent primary carcinomas and from cervical lymphnode metastases. Irradiated but macroscopically normal mucosa from a distant resection line, and uninvolved lymph nodes, served as control tissues. In 14 patients blood from the antecubital vein and the internal jugular vein was sampled at the time of surgery, for prostaglandin assay. Swabs for routine bacteriological culture were taken from all excised tumours. A part of each specimen taken for prostaglandin assay, and the remainder of the surgical specimen, were examined by standard histopathological procedures.

Tissues for assay were collected in dry, sterile containers and sent to the laboratory within 1 h of removal. They were then cut into small pieces with scissors and washed with Krebs' solution. Weighed samples were homogenized either in Krebs' solution to estimate prostaglandin-synthesizing ability, or in acidified aqueous ethanol to determine "basal" prostaglandins (Bennett *et al.*, 1973). Amounts synthesized from endogenous prostaglandin precursors released during homogenization were estimated by subtracting "basal" amounts from the "total" extracted from the homogenate in Krebs' solution (Unger *et al.*, 1971). The extracted material was assayed against $PGE_2$, using the rat gastric fundus strip preparation treated with various antagonists which increase the selectivity and sensitivity of the assay (Bennett *et al.*, 1973). Tentative characterization of the prostaglandin-like material extracted from 14 tumour homogenates was made by chromatography, using paper impregnated with silica gel, and the solvent system for group separation of PGE and PGF compounds (Stamford & Unger, 1972). Samples of venous blood were collected in lithium heparin tubes containing indomethacin (final concentration 10 μg/ml blood) and the prostaglandins extracted from the plasma (Unger *et al.*, 1971). The assay results are expressed as ng $PGE_2$ equivalents/g fresh tissue (median and semiquartile ranges) and analysed statistically by the Mann-Whitney U test or by Spearman's rank correlation.

FIG. 1.—Amounts of "total" prostaglandin-like material (ng $PGE_2$ equivalent/g on the vertical axis) extracted from residual or recurrent primary squamous carcinomas were significantly higher than from nodal metastases or uninvolved mucosa and lymph nodes.

### RESULTS

Seventy assays of prostaglandin-like material were made on 33 primary squamous carcinomas, 9 lymphnode metastases and 28 control specimens of uninvolved mucosa and lymph nodes. All the tissues had been previously irradiated, and those which were infected (as shown by swab cultures) were excluded. The amounts of "total" prostaglandin-like activity extracted from homogenates in Krebs's solution in these 3 groups are summarized in Fig. 1, expressed as median $PGE_2$ equivalents/g fresh tissue with semiquartile ranges in parentheses. The amounts extracted from primary squamous carcinomas (108(40–195)ng $PGE_2$ equivalents/g) were significantly higher than from nodal metastases (18(6–35)ng; $P = 0{\cdot}009$) or from uninvolved mucosa and lymph nodes (32(8–75)ng; $P = 0{\cdot}006$). The amounts from nodal metastases and uninvolved lymph nodes or mucosa were not significantly different ($P = 0{\cdot}4$).

Tumour site, size, degree of differentiation, necrosis, inflammation and fibrosis were examined by one of us (RLC) without prior knowledge of the prostaglandin results. Tumour total prostaglandins correlated with the extent of necrosis, inflammation and fibrosis, each scored on an arbitrary 1–3 point system (see Fig. 2). and they tended to correlate inversely with the interval between excision and radiotherapy with or without chemotherapy ($P = 0{\cdot}06$). More primary tumours removed within 3 months of radiotherapy with or without chemo therapy yielded total prostaglandin-like material of >200ng $PGE_2$ equivalents/g fresh tissue than did primary tumours removed after longer intervals ($P = 0{\cdot}04$). No association was detected between total prostaglandins and the tumour site, size or degree of differentiation. High amounts of prostaglandin-like activity (total amounts 72(22–118)ng $PGE_2$ equivalents/g) were extracted from many tumours showing direct invasion of bone or ossified laryngeal cartilage with marked osteoclastic activation, but these amounts were not significantly different from tumours invading soft tissues. In a relatively short postoperative follow-up of 3–21 months, total prostaglandin amounts from primary or metastatic tumours did not correlate with patient survival time.

FIG. 2.—Amounts of "total" prostaglandin-like material (ng $PGE_2$ equivalent/g on the vertical axis) extracted from residual or recurrent primary squamous carcinomas (●) and from nodal metastases (○) correlate with morphological scores of necrosis, inflammation and fibrosis (arbitrary units 1–9; see text).

High yields of prostaglandin-like material were also associated with local necrosis, inflammation and fibrosis in some control tissues. Two uninvolved lymph nodes with the high amounts of 420 and 460 ng $PGE_2$ equivalents/g showed intense reactive hyperplasia with sinus histiocytosis; both nodes were enlarged and had been thought clinically to contain metastatic carcinoma. Two specimens of macroscopically normal mucosa yielding 150 and 200 ng $PGE_2$ equivalents/g were inflamed, and one showed marked epithelial dysplasia.

Sufficient material was available to measure "basal" amounts of prostaglandins, and therefore "synthesized" amounts (total—basal) in 12 primary tumours, 5 nodal metastases and 4 specimens of uninvolved mucosa. "Total" amounts of tumour prostaglandins were high when "synthesized" amounts were high, and *vice versa* (correlation $P < 0{\cdot}001$) but there were insufficient data to assess the relationship in uninvolved tissues.

Chromatography of the "total prostaglandin" in homogenates of 14 recurrent carcinomas indicated the presence of several prostaglandins in varying amounts, running with $PGE_1$, $PGE_2$, $PGE_3$, $PGF_{1\alpha}$, $PGF_{2\alpha}$ and $PGF_{3\alpha}$. $PGE_2$-like material was present in all the extracts, with amounts ranging from 15% to 98% (median 60%) of the total biological activity assessed by the rat fundus-strip assay.

There was no consistent difference between the amounts of prostaglandins extracted from the peripheral venous blood (antecubital vein) of 12 patients and the blood draining the tumour-bearing area (internal jugular vein).

### DISCUSSION

The methods used here do not permit exact identification and quantitation of the individual prostaglandins. Indeed, there is no technique available which can do this with all the lipid derivatives in the small amounts of tumour available for study. Our bioassay measures the ability of relatively stable extracted lipids to contract rat gastric fundus, but underestimates substances with low biological activity on this tissue. Clearly the amounts of assayed biological activity are higher in extracts of treated primary squamous carcinomas of the head and neck than of nodal metastases or normal tissue. The morphological findings indicate that these high amounts of prostaglandins correlate with necrosis, inflammation and fibrosis in and around the primary tumour rather than with more specific attributes of the tumour itself, such as its site, size and degree of differentiation. Necrosis, inflammation and related changes are likely to be more pronounced after irradiation and chemotherapy, and total amounts of prostaglandins were often higher in tumours removed within 3 months of such treatment. Various forms of local damage, including ionizing radiation (Eisen & Walker, 1976) can increase the amounts of prostaglandin in tissue.

Much of the prostaglandins extracted from treated primary squamous carcinomas of the head and neck may reflect the activity of *host* stromal and inflammatory cells—particularly macrophages (Myatt *et al.*, 1975; Humes *et al.*, 1977; Carter, 1978; Bennett, 1979)—rather than the tumour cells themselves, though squamous-carcinoma cells presumably synthesize some prostaglandins. This might explain the unexpected finding of different total amounts of prostaglandins in extracts of primary squamous carcinomas and cervical-node metastases. Necrosis and subacute inflammation are frequently marked in primary squamous carcinomas of the head and neck, and such changes are exacerbated during and immediately after irradiation and chemotherapy. Metastatic squamous carcinoma in lymph nodes often becomes necrotic, but local inflammation is uncommon unless a nodal mass fungates and becomes secondarily infected; none of the tumours in this study was overtly infected. The occasional high amounts of prostaglandin-like material extracted from uninvolved control tissues (Fig. 1) were attributed either to subacute inflammation or, in the case of the lymph nodes, to marked sinus histiocytosis.

There was no evidence of prostaglandin release from the tumours into the blood. If prostaglandin E or F compounds had been released, the levels of PG-like material in plasma from the internal jugular vein would be higher than in peripheral venous plasma since most of the prostaglandin E or F compounds liberated from the tumour would be inactivated in the pulmonary circulation. On the other hand, any prostaglandin released from these treated tumours would inevitably be diluted by blood from other regions of the head and neck, and may therefore have been undetectable in our bioassay. Many breast tumours release prostaglandin-like material into the blood (Stamford *et al.*, 1979) and recalculation of data from a previous report (Mortel *et al.*, 1977) suggest that prostaglandins are released from some pelvic carcinomas (Bennett, 1979). In addition, recent work from our department (Tanner *et al.*, unpublished) has shown an association between increased levels of prostaglandin-like material in peripheral venous plasma and the degree of radiation-induced mucositis, a complication which severely limits effective radiotherapy.

Since all the tumours examined here had been previously treated, it is not appropriate to compare the results with those obtained with untreated human tumours of other types. This may explain the absence of a correlation between tumour prostaglandins and patient survival (*cf.* Bennett *et al.*, 1979) though the numbers of patients here are small. Nevertheless, prostaglandins associated with osteolytic primary squamous carcinomas of the head and neck may facilitate the direct spread of strategically placed tumours to adjacent bone or ossified cartilage (Carter *et al.*, 1980; Carter & Tanner, 1979).

Several studies in laboratory animals have demonstrated benefits of using prostaglandin synthesis inhibitors in the treatment of malignant disease (Bennett, 1979), and a trial is now in progress at King's College Hospital to evaluate flurbiprofen in the clinical management of patients with advanced (T3, T4) squamous carcinomas of the head and neck who are initially treated by radiotherapy (with or without chemotherapy) before planned radical surgery.

We are indebted to Mr Peter Clifford, F.R.C.S., Mr H. J. Shaw, F.R.C.S., Dr V. M. Dalley and Dr A. D. O'Connor for aceess to clinical material. N.S.B.T. was supported by a Royal Marsden Hospital Clinical Research Fellowship, and I.F.S by the Medical Research Council. Miss R. Jiwa and Mrs J. E. Wright, supported by the Cancer Research Campaign, gave technical assistance.

### REFERENCES

ATKINS, D., IBBOTSON, K. J., HILLIER, K., HUNT, N. H., HAMMONDS, J. C. & MARTIN, T. J. (1977) Secretion of prostaglandins as bone-resorbing agents by renal cortical carcinoma in culture. *Br. J. Cancer*, **36**, 601.

BENNETT, A. (1979) Prostaglandins and cancer. In *Practical Applications of Prostaglandins and their Synthesis Inhibitors*. Ed. S. M. M. Karin. Lancaster: MTP Press. p. 149.

BENNETT, A., BERSTOCK, D. A., RAJA, B., STAMFORD, I. F. (1979) Survival time after surgery is inversely related to the amounts of prostaglandin extracted from human breast cancers. *Br. J. Pharmacol.*, **66**, 451P.

BENNETT, A., CHARLIER, E. M., MCDONALD, A. M., SIMPSON, J. S. & STAMFORD, I. F. (1976) Bone destruction by breast tumours. *Prostaglandins*, **11**, 461.

BENNETT, A., MCDONALD, A. M., SIMPSON, J. S. & STAMFORD, I. F. (1975) Breast cancer, prostaglandins and bone metastases. *Lancet*, i, 1218.

BENNETT, A., STAMFORD, I. F. & UNGER, W. G. (1973) Prostaglandin $E_2$ and gastric acid secretion in man. *J. Physiol.*, **229**, 349.

BENNETT, A., DEL TACCA, M., STAMFORD, I. F. & ZEBRO, T. (1977) Prostaglandins from tumours of human large bowel. *Br. J. Cancer*, **35**, 881.

CARTER, R. L. (1978) Metastatic potential of malignant tumours. *Invest. Cell Pathol.*, **1**, 275.

CARTER, R. L. & TANNER, N. S. B. (1979) Local invasion by laryngeal carcinoma—the importance of focal (metaplastic) ossification within laryngeal cartilage. *Clin. Otolaryngol.*, **4**, 283.

CARTER, R. L., TANNER, N. S. B., CLIFFORD, P. & SHAW, H. J. (1980) Direct bone invasion in squamous carcinomas of the head and neck: pathological and clinical implications. *Clin. Otolaryngol.* (in press).

EISEN, V. & WALKER, D. I. (1976) Effect of ionizing radiation on prostaglandin-like activity in tissues. *Br. J. Pharmacol.*, **57**, 527.

GALASKO, C. S. B. (1976) Mechanisms of bone destruction in the development of skeletal metastases. *Nature*, **263**, 507.

GALASKO, C. S. B. & BENNETT, A. (1976) Relationship of bone destruction in skeletal metastases to osteoclast activation and prostaglandins. *Nature*, **263**, 508.

HUMES, J. L., BONNEY, R. J., PELUS, L. & 4 others (1977) Macrophages synthesise and release prostaglandins in response to inflammatory stimuli. *Nature*, **269**, 149.

MORTEL, R., ALLEGRA, J. C., DEMERS, L. M. & 6 others (1977) Plasma prostaglandins across the tumor bed of patients with gynecologic malignancy. *Cancer*, **39**, 2201.

MYATT, L., BRAY, M. A., GORDAN, D. A. & MORLEY, J. (1975) Macrophages on intrauterine contraceptive devices produce prostaglandins. *Nature*, **257**, 227.

O'CONNOR, A. D., CLIFFORD, P., DURDEN SMITH, D. J., EDWARDS, W., HOLLIS, B. A. & DALLEY, V. M. (1977) Synchronous VBM and radiotherapy in the treatment of squamous carcinoma of the head and neck. *Clin. Otolaryngol.*, **2**, 347.

POWLES, T. J., CLARK, S. A., EASTY, D. M., EASTY, G. C. & NEVILLE, A. M. (1973) The inhibition by aspirin and indomethacin of osteolytic tumour deposits and hypercalcaemia in rats with Walker tumour and its possible application to human breast cancer. *Br. J. Cancer*, **28**, 316.

POWLES, T. J., DOWSETT, M., EASTY, D. M., EASTY, G. C. & NEVILLE, A. M. (1976) Breast cancer osteolysis, bone metastases and antiosteolytic effects of aspirin. *Lancet*, i, 608.

PRICE, L. A. & HILL, B. T. (1977) A kinetically based approach to the chemotherapy of head and neck cancer. *Clin. Otolaryngol.*, **2**, 339.

STAMFORD, I. F., MACINTYRE, J. & BENNETT, A. (1980) Human breast cancers release prostaglandins into the blood. In *Advances in Prostaglandins and Thromboxane Research*. Eds. Samuelsson, Ramwell & Paolett. New York: Raven Press. p. 571.

STAMFORD, I. F. & UNGER, W. G. (1972) Improved purification and chromatography of extracts containing prostaglandins. *J. Physiol.*, **225**, 4P.

TASHJIAN, A. H., VOELKEL, E. F., GOLDHABER, P. & LEVINE, L. (1974) Prostaglandins, calcium metabolism and cancer. *Fed. Proc.*, **33**, 81.

UNGER, W. G, STAMFORD, I. F. & BENNETT, A. (1971) Extraction of prostaglandins from human blood. *Nature*, **233**, 336.